# The Role of Proteases and Serpin Protease Inhibitors in β-Cell Biology and Diabetes

**DOI:** 10.3390/biom12010067

**Published:** 2022-01-02

**Authors:** Yury Kryvalap, Jan Czyzyk

**Affiliations:** Department of Laboratory Medicine and Pathology, University of Minnesota, 420 Washington Ave. SE, Minneapolis, MN 55455, USA; ykryvala@umn.edu

**Keywords:** diabetes, pancreatic islet, protease, serpin

## Abstract

Regulation of the equilibrium between proteases and their inhibitors is fundamental to health maintenance. Consequently, developing a means of targeting protease activity to promote tissue regeneration and inhibit inflammation may offer a new strategy in therapy development for diabetes and other diseases. Specifically, recent efforts have focused on serine protease inhibitors, known as serpins, as potential therapeutic targets. The serpin protein family comprises a broad range of protease inhibitors, which are categorized into 16 clades that are all extracellular, with the exception of Clade B, which controls mostly intracellular proteases, including both serine- and papain-like cysteine proteases. This review discusses the most salient, and sometimes opposing, views that either inhibition or augmentation of protease activity can bring about positive outcomes in pancreatic islet biology and inflammation. These potential discrepancies can be reconciled at the molecular level as specific proteases and serpins regulate distinct signaling pathways, thereby playing equally distinct roles in health and disease development.

## 1. Introduction

Protease activity is critical for the survival of multicellular organisms. Proteases break down peptide bonds, resulting in irreversible posttranslational protein modifications. The complement and clotting systems are examples of proteolytic cascades which, if inadequately activated, can place the host at risk of serious health problems associated with an increased susceptibility to infection and bleeding disorders. It is not surprising, therefore, that proteases are modulated by a number of inhibitors, which may themselves be regulated in turn. One group of evolutionary conserved protease inhibitors are the serine protease inhibitors, also known as serpins [1].

**Figure 1:**
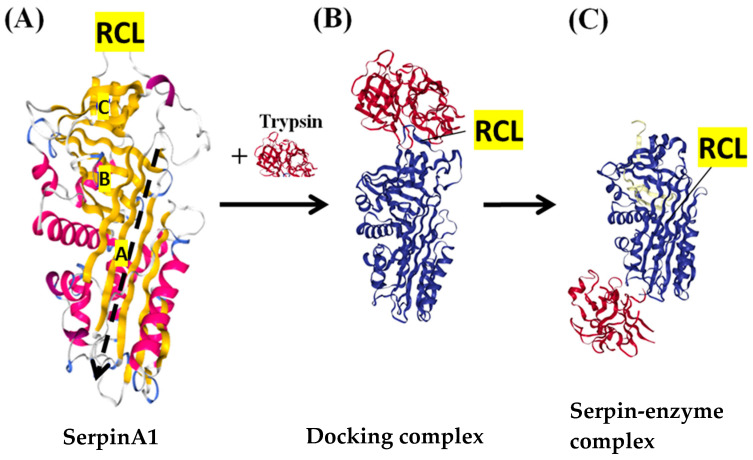
The structure and mechanism of inhibitory serpins. (**A**) The native structure of the archetypal serpin—serpinA1 (protein data bank (PDB) code 1QLP). The α-helices are shown in pink, and 3 β-sheets with gold color are marked with A, B and C. The reactive center loop (RCL) is at the top of the molecule and between A sheet and C sheet. The black dashed line indicates the path of RCL insertion after RCL is cleaved by the target protease. (**B**) The docking complex between serpinA1 (blue) and inactive trypsin (red) (PDB code 1OPH). The protease docked onto the RCL. (**C**) The final serpin–enzyme complex (PDB code 1EZX). After the RCL is cut, the serpin undergoes a transition from the stressed form to the relaxed form, and the docking complex becomes the serpin–enzyme complex, in which the distorted protease hangs at the base of the serpin molecule.

In eucaryotes, members of the serpin family can be subdivided into 16 ‘clades’ according to common ancestries, amino acidic sequences, and function [1,2,3,4]. Notably, all serpins share a conserved tertiary structure, consisting of three β-sheets (A, B, and C), seven to nine α-helices, and a reactive center loop (RCL), which is approximately 17 amino acids and tethered between the A and C β-sheets [1]. Figure 1A shows the structure of the prototype serpin, serpin A1 [5]. Serpin specificity and function is defined by its RCL, as the amino acid sequence of the RCL is specifically recognized by the target protease. When a serpin molecule binds to its target protease, the RCL is cleaved by the active site of the protease, which results in a conformational shift of the serpin from the ‘stressed’ to the ‘relaxed’ form. As a result of this conformational change, the protease becomes trapped in a serpin–enzyme complex (Figure 1B,C) [2,6]. Therefore, the inhibitory mechanism of serpins is ‘suicidal’, and each serpin molecule is ‘single use’ [7].

Most serpins inhibit serine proteases, although serpins that inhibit caspases and papain-like cysteine proteases have also been identified [1,2]. Moreover, certain serpins lack protease inhibitor characteristics. The primary function of inhibitory serpins is to regulate proteolytic cascades and protect cells from nonspecific protease-mediated damage [4]. For example, expression of serpinA1 (α1-antitrypsin) in the circulation is induced during inflammation to prevent damage in the respiratory tract [8]. The noninhibitory serpins perform diverse functions, including storage, regulation of blood pressure, and serving as molecular chaperones.

Serpins are also implicated in a number of diseases. Serpinopathies are caused by mutations or dysfunction of serpins that form polymer aggregates and ultimately result in tissue damage [2,3]. Two mechanisms by which serpinopathies harm the body have been proposed. This first is the accumulation of polymers that induce endoplasmic reticulum stress and inflammation, e.g., liver cirrhosis due to mutated α1-antitrypsin precipitates [9]. The second is the loss of serpin function resulting in uncontrolled protease activity, e.g., emphysema caused by overactivation of elastase due to α1-antitrypsin deficiency [10]. Acquired C1 inhibitor deficiency is another example of uncontrolled protease activity secondary to autoantibodies against the C1 inhibitor (serpin G1; C1-INH). Blocking C1-INH function activates the classic pathway of the complement system, which eventually leads to angioedema [11].

Serpins in the clade B subfamily are also termed ov-serpins because chicken ovalbumin is the archetypal member of this group [12]. There are 13 clade B serpins in humans, and most of them, apart from serpinB5 and serpinB11, have inhibitory functions (Table 1). Among the inhibitory clade B serpins, serpinB4 and serpinB9 are cross-class serpins, which means they can block serine proteases and other protease types, while serpinB3 and serpinB13 selectively inhibit cysteine proteases [7,13]. The primary function of these inhibitory clade B serpins appears to be cellular protection against proteases released from activated immune cells or from the lysosome [4,14,15,16].

Most serpins are extracellular molecules, yet the clade B serpins are mainly retained in the cell due to their lack of an N-terminal secretory signal [7,12]. Nevertheless, clade B serpins can be exposed to the environment via leakage from damaged tissue or in other settings [12]. For example, membrane-bound serpinB3 has been detected on the surface of mononuclear cells [17], and serpin B1 has been reported to be secreted from the liver to promote pancreatic β-cell proliferation [18]. In our studies, we found that some (but not all) nonobese diabetic (NOD) animals produced autoantibodies against serpinB13 [19], suggesting that serpinB13 was exposed to the extracellular environment to provoke this antibody response. In another study, we also noticed that serpinB13 was able to reach the extracellular milieu during the culture of mouse embryonic pancreas explants [20]. Finally, a study demonstrating that serpinB13 functions in the extracellular matrix to suppress angiogenesis further supports the idea that clade B serpins can be released from the cell under certain conditions [21]. The limited extracellular presence of clade B serpins positions them to fine tune the activity of cellular protease that may also have reached the extracellular compartment. This delicate balance between proteases an antiproteases at the level of pancreatic islets provides important, and largely underappreciated, cues for the development and regeneration of endocrine cells in the pancreas as well as their ability to respond to inflammation.

## 2. Preclinical and Clinical Attempts to Treat Type 1 Diabetes with Proteases

The idea that protease activity may be exploited for therapeutic purposes in autoimmune diabetes is based on observations that intravenous, or oral, application of proteolytic enzymes ameliorates immune-mediated diseases, including animal models of immune complex glomerulonephritis [22,23], allograft arteriosclerosis [24], and multiple sclerosis [25]. Phlogenzym—a mixture consisting of the hydrolytic enzymes trypsin and bromelain and the antioxidant rutoside—was used in some of these studies and reduced the severity of disease. The presumptive mechanism was that T cell activation may be affected by changes in surface molecule expression. In a similar fashion, NOD female mice fed with Phlogenzym during the subclinical phase of diabetes, e.g., from 6 to 10 weeks of age, showed a deceleration of diabetes onset [26]. Examination of autoreactive T cell clones isolated from prediabetic subjects, and patients with a recent onset of type 1 diabetes (T1D), revealed that treatment of these cells with several distinct proteases (e.g., trypsin, papain, chymotrypsin, or bromelain) decreased expression of CD3 and CD44. A similar treatment of B lymphocytes and immature dendritic cells either decreased or upregulated expression of HLA-DR, CD54, CD58, and CD80 [27]. These changes coincided with impaired T cell proliferative activity, as well as downregulation of proinflammatory cytokine interferon-gamma. These observations indicate that protease treatment modulates cell surface expression of homing receptors, costimulatory molecules, and receptors involved in antigen epitope presentation and recognition. In doing so, cytokine production is skewed from a Th1 to a noninflammatory Th2 profile, which may explain the overall anti-inflammatory effect of these proteases in T1D and other immunological diseases. Studies from our laboratory are in support of the notion that protease activity in pancreatic islets helps to resolve inflammation via cleavage of the extracellular domain of CD19 in B cells and CD4 in T cells ([28], see below). The in vitro and preclinical studies mentioned above prompted the launch of a small clinical trial involving oral treatment of proteases/flavonol in subjects at risk for T1D [29]. This treatment was safe and well tolerated but did not significantly prevent diabetes, although there seemed to be a trend in favor of the delay in diabetes onset in the group treated with proteases compared with placebo-treated controls during eight observational years. 

## 3. Preclinical and Clinical Attempts to Treat Type 1 Diabetes with Antiproteases

Alpha-antitrypsin (AAT), also known as alpha1-protease inhibitor (α_1_-PI), is encoded in humans by the *SERPINA1* gene. It is one of the most abundant serine protease inhibitors produced and secreted by the liver. It inhibits the proteolytic enzymes neutrophil elastase, trypsin, cathepsin G, and proteinase 3, although new findings have emerged on the ability of AAT to also inhibit other classes of proteases, such as metalloproteases and cysteine-aspartic proteases [8]. Congenital AAT deficiency is associated with the development of pulmonary emphysema and hepatic cirrhosis due to defective confinement of proteolytic activity in the lung and accumulation of an abnormal form of AAT in hepatocytes, respectively.

Although several early studies on AAT indicated increased levels of this protease inhibitor in the sera of diabetic subjects [30,31,32], subsequent observations have not confirmed these findings and in fact suggest decreased serum concentrations of AAT and increased plasma serine proteinase in insulin dependent diabetes. These changes were observed in both sexes, although males with onset of diabetes at an age less than 15 years were the most affected [33]. Thus, diabetes, early onset of the disease, and male sex appear to be associated with alterations in the protease–antiproteinase balance in type 1 diabetics. Glucose, per se, may also be a factor contributing to the lower AAT, as adding glucose in vitro significantly reduces the levels of purified AAT and that of AAT in serum [34]. The clinical significance of these observations remains unclear. However, the notion that elastase, the activity of which is largely controlled by AAT, is a major phagocytic lysosomal protease released into the extracellular space during inflammation suggests that abnormalities in the normal levels of protease inhibitors may contribute to a defective resolution of the inflammatory response and tissue healing, which is observed in long-standing diabetic patients.

Direct evidence that inhibition of AAT is protective in T1D was shown in studies using a recombinant adeno-associated viral system to deliver and express the AAT gene [35,36]. Using this system, NOD mice overexpressing human AAT were shown to have diminished insulitis and, depending on the efficiency of gene delivery, were either partially, or nearly completely, protected from developing diabetes. Moreover, according to another study, administering a short course of purified human AAT into NOD mice with new-onset diabetes, and which were treated with insulin, resulted in marked restoration of pancreatic β-cell mass and euglycemia, compared with mice that were treated with insulin alone [37]. Based on these, and several other studies, it has been proposed that both the anti-inflammatory properties [38,39] and the ability to protect pancreatic β cells from apoptosis [40] may be responsible for AAT’s antidiabetic effect. These features may also explain AAT’s potential to prolong pancreatic islet allograft survival, which has been described by several groups [41,42,43,44]. The abovementioned effects, along with a high safety profile that is associated with multiple intravenous doses of AAT in humans [45], provided the rationale to design and execute a randomized double-blind study in children with recent onset T1D. Although this intervention trial failed to demonstrate a significant positive impact on blood glucose control and residual β-cell function, a subgroup analysis revealed certain benefits in adolescent patients receiving the high dosage of AAT (120 mg/kg) [46].

## 4. The Role of Proteases Inhibited by Clade B Serpins in Diabetes 

Cathepsin L (catL) is a major lysosomal cysteine protease that is important for antigen presentation in macrophages and cortical epithelia cells, where it is responsible for degradation of the invariant chain [47]. CatL defect in the thymus results in impaired positive selection of CD4^+^ T cells and an increased number of the peripheral regulatory T (Treg) cells with anti-inflammatory properties. Consequently, an enriched Foxp3^+^ Treg population (rather than defective development of diabetogenic T cells in the thymus) completely protects NOD mice with catL deficiency from insulitis and diabetes [48]. The genetic deficiency of other important lysosomal cysteine proteases, cathepsin S (catS) and B (catB), results in only partial protection from autoimmune diabetes in NOD mice by the age of six months [49]. The reason for this incomplete penetrance is unclear. Likewise, whether diabetes incidence would change further in older NOD mice with knockout of the catS or catB genes remains unknown. Of note, catS and cathepsin C and W were found to be upregulated at sites of leukocyte penetration of the peri-islet capsule, and at these sites were associated with loss of peri-islet basement membrane and intracellular matrix components [50]. These observations suggest that controlled inhibition of protease activity may help to prevent destructive infiltration of the islets by inflammatory cells during the development of autoimmune diabetes.

Additional studies with catL inhibitor (Clik148) in a model of cyclophosphamide-induced diabetes in NOD mice confirmed the genetic knockout studies indicating that specific inhibition of this protease affords strong protection from disease and insulitis, albeit via a mechanism that may not involve Treg cells. In these studies, the authors showed that there was no increase in the number of Treg cells following the administration of Clik148. Instead, they noticed that enhanced activation of CD8^+^ (not CD4^+^) T cells isolated from the pancreatic lymph nodes was suppressed by the catL inhibitor [51]. Moreover, surface expression of class II molecules induced by IFN-γ and TNF-α stimulation in the mouse islet β-cell line (Min6) was inhibited following incubation with Clik148 but not with inhibitors of catS or catB. This implies that inhibition of catL may play a role in preventing autoantigen presentation to CD4^+^ T cells [52].

Another potential mechanism for the influence of catL on autoimmune diabetes development is indirectly implicated through a study showing that this protease regulates the differentiation of T helper 17 (Th17) cells [53]. Since Th17 cells have been implicated both in the pathogenesis of, and protection from, T1D [54,55], suppressing these cells with catL inhibitors (e.g., serpinB1 or a chemical compound) may affect disease development, including potential acceleration of diabetes clinical onset. This is in contrast to the widely held concept that proteases have primarily detrimental effects in T1D and other autoimmune diseases, although one study suggested that the catL gene belongs to a group of the 100 ‘protective genes’ in NOD mice [56]. Our studies implicated catL expressed in the pancreatic ductal epithelium in islet upregulation of Reg genes, which have been linked to enhanced regeneration of pancreatic tissue. This suggests that catL may regulate functional crosstalk between the exocrine and endocrine pancreas to enhance regenerative or healing outcomes [57]. Overall, studies on catL suggest a complex role in diabetes. While expression of catL in cells of the immune system may elicit T cell priming against islet autoantigens, its expression at a local level in pancreatic tissue may improve certain aspects of islet biology and be protective against diabetes.

## 5. The Role of Clade B Serpins in Diabetes 

SerpinB1 is a member of the clade B serpins that inhibits the serine proteases elastase, cathepsin G, and proteinase-3 [58]. SerpinB1-deficient mice have considerably increased mortality relative to wild-type mice, in association with late-onset, failed bacterial clearance in the experimental model of *Pseudomonas aeruginosa* lung infection [59]. Under normal conditions, serpinB1 is highly expressed in neutrophils. However, under conditions of insulin resistance in the setting of hepatocyte-specific knockout of insulin receptor in mice or in the setting of obesity and type 2 diabetes in humans, this serpin demonstrated an augmented expression in the liver and serum [18,60]. Based on these observations and additional experiments with recombinant serpinB1 and small-molecule compounds that mimic the inhibitory function of serpinB1 against elastase [18], serpinB1 released from the liver has been proposed to stimulate proliferation of insulin-producing cells and contribute to the pancreatic islet hyperplasia that is frequently observed in diabetic patients. This may also apply to pancreatic β-cell proliferation in the Zebrafish system with overexpressed serpinB1. Another study, however, suggested that protease molecules, rather than their inhibitors, control regeneration of pancreatic endocrine cells in this animal model [61]. Of note, genotyping for SERPINB1 gene polymorphism, namely SERPINB1 rs15286, which is a transition A/G SNP in the 3′ UTR region of the SERPINB gene, revealed the AA genotype that is associated with an overall better glycemic control and better pancreatic β-cell function in the Egyptian type 2 diabetic patients [62]

SerpinB8 is another member of the clade B serpin family, which is expressed in several tissues including β-cells [63,64]. It is an immunohistochemical marker for neuroendocrine tumors in the pancreas [65], and although its physiological role is largely unknown, in vitro studies suggest that it is an inhibitor of the ubiquitously expressed furin-like prohormone convertase [66]. Furin, which is also highly expressed in insulin-producing cells, has been shown to control the growth and differentiation of several pancreatic β-cell lines [67]. At the molecular level, furin has been proposed to cleave Ac45, an accessory subunit of the proton pump V-ATPase, which is important for acidification during β-cell granule maturation and conversion from proinsulin to insulin. Consequently, furin knockout mice demonstrate impaired insulin content and secretion [68]. These animals also exhibit abnormal processing of insulin receptor, decreased pancreatic β-cell mass, and proliferation, and ultimately develop progressive, age-dependent glucose intolerance [69,70]. Although the data available on serpinB8 are scant, the restrictive pattern of this serpin’s expression in the pancreas may reflect the function of this molecule to inhibit furin in the islets, thereby implicating it in various aspects of islet biology.

Studies from our laboratory have focused on serpinB13, which inhibits catL and cathepsin K [71,72]. In the pancreas, serpinB13 is expressed in the exocrine ducts [28,57]. Our initial studies found a novel autoantibody that binds serpinB13 and plays a protective role in diabetes [19]. Later studies with a monoclonal antibody to serpinB13 revealed that this protection may be caused by impeding serpinB13 from neutralizing catL and subsequent upregulation of the cleavage of key cell surface molecules in lymphocytes that accumulate in the pancreatic islets of NOD mice [28]. The net outcome of these changes is impaired leukocyte function and reduced severity of autoimmune inflammation. Notwithstanding the anti-inflammatory impact of anti-serpin immunity, serpinB13 antibody also stimulates regenerative changes in the islets. More specifically, injecting mice with a monoclonal antibody to serpinB13 (which we used as a model), stimulates the proliferation of insulin-producing cells in the islets, significantly increases the number of pancreatic islets per animal, and ultimately leads to an increase in the pancreatic β-cell mass [20,56,73]. Moreover, the generation of additional endocrine progenitor cells with Ngn3 expression was found in the pancreas of mouse embryos exposed to inhibition of serpinB13 with chemical inhibitors or antibody to serpinB13. This upregulation was linked to the catL-mediated cleavage of the extracellular domain of the Notch1 receptor and inhibition of the Notch signaling pathway, which has been heavily implicated in pancreatic β-cell development [20]. The relevance of studies on serpinB13 in mice to humans was underscored by a finding that children with recent onset T1D and who are positive for serpinB13 AA progress to clinically visible pancreatic β-cell defect at a slower rate compared with T1D patients that are negative for this AA.

Although it remains unclear how exactly the antibody-mediated immunological response to serpinB13 in pancreatic ducts controls development, biology, and inflammation in pancreatic islets, there are clues highlighted by our in vitro observations. Namely, in the presence of an extract of pancreatic ductal epithelium and a mAb to serpinB13, human islets markedly upregulate genes that have been implicated in pancreatic β-cell regeneration [57]. Remarkably, this effect was not observed when the islets were co-incubated with extracts of nonductal exocrine pancreatic epithelium. Thus, the effect of the serpin antibody on islets is indirect and consistent with the notion of islet regulation by surrounding tissues.

## 6. Conclusions

Proteases and antiproteases play diverse roles in normal islet biology and diabetes. Although some studies suggest that protease activity aids inflammatory cell penetration of the pancreatic islets and restricts pancreatic β-cell proliferation, other studies suggest that certain proteases improve islet biology and pancreatic β-cell development, thereby delaying the onset of diabetes. Moreover, the proteases that influence the islets are expressed in the exocrine pancreas, suggesting a functional crosstalk between distinct tissue compartments in the pancreas and the role that these relationships may play in diabetes. Additional research should bring a better understanding of the molecular events that are responsible for protease-induced changes in pancreatic islets and how signals within these network pathways can be enhanced or suppressed for the purpose of developing novel therapeutic interventions in diabetes.

## Figures and Tables

**Table 1:** Inhibitory function and targets of human clade B serpins.

Serpin	Alternative Name	Inhibitory Function	Potential Protein Targets
SerpinB1	PI-2, Neutrophil elastase inhibitor	+	Elastase, Chymotrypsin, Cathepsin G, Protease 3
SerpinB2	PAI-2	+	uPA, tPA
SerpinB3	SCCA1	+	Papain, Cathepsin L, K, S
SerpinB4	SCCA2, Leupin	+	Cathepsin G, Chymase
SerpinB5	Maspin	−	-
SerpinB6	CAP1, PI6	+	Thrombin, Trypsin, Factor Xa, Cathepsin G, u-PA
SerpinB7	Megsin	+	Plasmin
SerpinB8	CAP2, PI8	+	Furin, Trypsin, Factor Xa, Thrombin, Chymotrypsin, Subtilisin A
SerpinB9	CAP3, PI9	+	Granzyme B, Subtilisin A, Caspase 1, 4, 8, 10
SerpinB10	Bomapin, PI10	+	Thrombin, Trypsin
SerpinB11	Epipin	-	-
SerpinB12	Yukopin	+	Trypsin, Plasmin
SerpinB13	Headpin, Hurpin	+	Cathepsin K, L, V

SCCA, Squamous cell carcinoma antigen; CAP, Cytoplasmic antiprotease; PI, Protease inhibitor; uPA, Urokinase plasminogen activator; tPA, Tissue plasminogen activator.

## Data Availability

Not applicable.
